# A New Nonlinear Photothermal Iterative Theory for Port-Wine Stain Detection

**DOI:** 10.3390/ijerph19095637

**Published:** 2022-05-05

**Authors:** Na Cao, Hongtao Liang, Ruoyu Zhang, Yanhua Li, Hui Cao

**Affiliations:** Shaanxi Key Laboratory of Ultrasound, School of Physics and Information Technology, Shaanxi Normal University, Xi’an 710062, China; caona@snnu.edu.cn (N.C.); li-yanhua@snnu.edu.cn (H.L.); zhangruoyu@snnu.edu.cn (R.Z.); wangyajun@cau.edu.cn (Y.L.)

**Keywords:** nonlinear thermal diffusion equation, new numerical iterative method, port-wine stain, sensitivity

## Abstract

The development of appropriate photothermal detection of skin diseases to meet complex clinical demands is an urgent challenge for the prevention and therapy of skin cancer. An extensive body of literature has ignored all high-order harmonics above the second order and their influences on low-order harmonics. In this paper, a new iterative numerical method is developed for solving the nonlinear thermal diffusion equation to improve nonlinear photothermal detection for the noninvasive assessment of the thickness of port-wine stain (PWS). First, based on the anatomical and structural properties of skin tissue of PWS, a nonlinear theoretical model for photothermal detection is established. Second, a corresponding nonlinear thermal diffusion equation is solved by using the new iterative numerical method and taking into account harmonics above the second-order and their effects on lower-order harmonics. Finally, the thickness and excitation light intensity of PWS samples are numerically simulated. The simulation results show that the numerical solution converges fasterand the physical meaning of the solution is clearerwith the new method than with the traditional perturbation method. The rate of change in each harmonic with the sample thickness for the new method is higher than that for the conventional perturbation method, suggesting that the proposed numerical method may provide greater detection sensitivity. The results of the study provide a theoretical basis for the clinical treatment of PWS.

## 1. Introduction

Port-wine stain (PWS), also known as nevus flammeus, is a congenital telangiectasia deformity. It is the most common type of benign vascular malformation and is difficult to cure [1,2]. Wine discoloration often occurs on the head, face, and neck, and severe cases are accompanied by overgrowth of soft tissues and bones in the lesion area, resulting in local enlargement and deformation [3]. These lesions greatly affect the patient’s appearance, decrease their quality of life, and cause considerable mental stress [4,5]. Therefore, early and effective intervention is particularly important.

Currently, the evaluation and prediction of PWS treatment consist of invasive and noninvasive approaches. Although biopsy has long been considered the gold standard for treatment, it is invasive and not widely performed. Noninvasive treatments include chromatography [6], dermoscopy [7], high−frequency ultrasound [8,9], and laser scatter imaging [10]. However, none of these commonly used imaging techniques provide adequate imaging depth and contrast to accurately assess PWS. A recent trial has shown that the use of photoacoustic techniques for the clinical evaluation of PWS disorders provides a new method for the quantitative evaluation of PWS [11].

Due to combining the advantages of both deep penetration provided by ultrasound imaging [12,13] and high contrast provided by optical imaging [14], photoacoustic technology [15,16] has become a research frontier and hot spot in the field of biomedical imaging. Most studies of photoacoustic techniques ignore the effect of the local temperature increase of the medium caused by light absorption on the thermodynamic parameters of the medium (e.g., thermal conductivity, density, and isobaric specific heat capacity) and assume that the thermodynamic parameters are constant. However, a statistically significant increase in the local temperature can change the values of thermodynamic parameters of the medium, and contribute to nonlinear photoacoustic conversion. The nonlinearity describing the thermal conductivity problem of laser irradiated tissue can be caused by various physical reasons, e.g., laser−induced formation of bubbles due to temperature dependence of gas solubility [17,18]; temperature dependence of thermodynamic parameters [19], etc. The nonlinear photoacoustic effect has attracted increasing attention as a possible means of selective detection of contrast agents by heat accumulation and local temperature increase thus enhancing the photoacoustic signal, and it is necessary to consider the nonlinearity of the thermal parameters in the thermal diffusion equation [20,21].

Therefore, this paper investigates the theory of thermal field imaging of PWS using nonlinear photoacoustic effect in the frequency domain by introducing nonlinear thermal conductivity coefficients. Based on previous work [22,23], a new semianalytic numerical iterative method is proposed in this paper. First, the temperature field is expanded in a Fourier series in the frequency domain to separate time variables and spatial coordinates. Then, the nonlinear diffusion equation is solved by selecting the appropriate high−frequency harmonics according to the specific requirements for calculation accuracy. Finally, the thickness and excitation light intensity are numerically simulated in light absorbers of different thicknesses using the new iterative numerical method and the conventional perturbation method. The results show that the solution bythe numerical method has greater sensitivity and bandwidth than that of the perturbation method and can better distinguish between PWS samples of different thicknesses. This work extends the application of nonlinear thermal field theory in clinical medicine and contributes to a better understanding of PWS in lesions during different stages of development.

## 2. Theoretical Analysis

### 2.1. Theoretical Model

**Figure 1:**
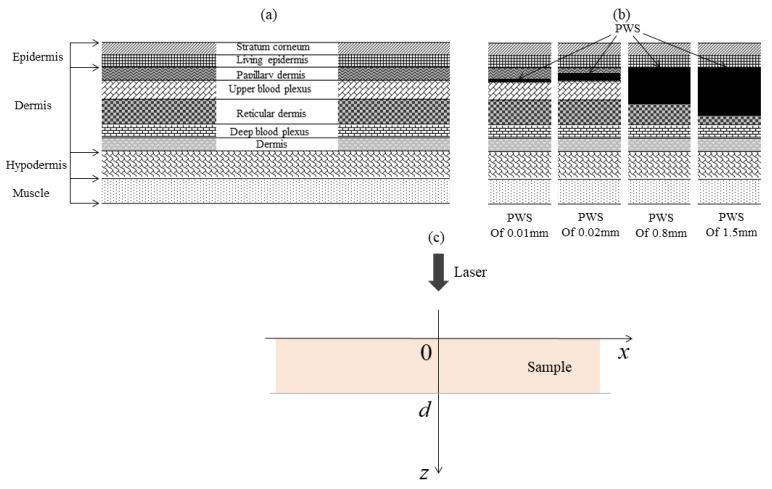
(**a**) Schematic of multilayered skin; (**b**) schematic of skin with different growth phases of a cancerous lesion; (**c**) theoretical model for photoacoustic detection of skin tissue.

The skin is the largest and most important tissue in the human body. Anatomically, the skin can be divided into the epidermis, dermis, hypodermis, and muscle layers, as shown in Figure 1a. The epidermis consists of the high-fat, low-water stratum corneum and the melanin-containing living epidermis. Similarly, the dermis has two sublayers: the papillary dermis and the reticular dermis, which contain two vascular plexuses; the upper and deep blood plexuses are located in the upper and lower reticular layers of the dermis. The subcutaneous tissue consists mainly of fat cells.

In human dermatology, PWS is one of the most common benign tumors involving vascular malformations, with an incidence of between approximately 0.3 and 0.5% in the general population. PWS are mainly located in the papillary layer of the dermis and the upper layer of the reticular layer, with a diameter of approximately 0.01~0.15 mm and a thickness of approximately 0.001~1.5 mm [24]. Lesions can expand in size to cover a larger dermal area over time, as shown in Figure 1b.

Based on the anatomical and structural properties of skin tissue lesions, this paper constructs a nonlinear photothermal detection model for PWS, as shown in Figure 1c. Based on the optical attenuation, imaging depth, and other information reported by Chen et al., a laser pulse with a wavelength of 840 nm was chosen for PWS detection in this study. The parameters of each skin layer at a wavelength of 840 nm are shown in Table 1 [11,25,26,27].

### 2.2. Nonlinear Thermal Diffusion Equation

A wave pulsed laser $I(t)=2\sqrt{\frac{\ln(2)}{\pi }}\frac{\omega_{P}}{\tau }\exp\{-4\ln(2){\left(\frac{t-t_{0}}{\tau }\right)}^{2}\}$ irradiates the sample, and light is absorbed by the sample and converted into heat, generating a photoacoustic signal through thermal expansion, where τ is the pulse width, $t_{0}$ is the pulse center, and $w_{P}$ is the luminous flux (mJ/cm^2^). The fundamental equation of the photoacoustic imaging theory is based on the thermal diffusion equation; therefore, the thermal diffusion equation and boundary conditions in the sample are [28,29]:

(1) $$T_{s,zz}(z,t)=\frac{1}{\alpha }T_{,t}(z,t)-\frac{Q(z,t)}{K_{s}},$$

(2) $$\left\{ \begin{array}{l} K_{s}T_{s,z}(0,t)=K_{g}T_{g,z}(0,t)-\beta^{\prime }w_{P}[1+\exp(j\omega t)]+H[T_{s}(0,t)-T_{g}(0,t)],z=0,\\ K_{s}T_{s,z}(d,t)=K_{g}T_{g,z}(d,t)-\beta^{\prime }(1-\beta^{\prime })w_{P}\exp(-\beta d)[1+\exp(j\omega t)]+H[T_{s}(d,t)-T_{g}(d,t)],z=d. \end{array}\right.$$

where $T_{s}(z,t)$, $T_{g}(z,t)$ are the temperature rise and fall of the sample and air, respectively, $\alpha =K_{s}/(\rho C_{P})$ is the thermal diffusion coefficient, ρ and $C_{p}$ are the density and isobaric specific heat capacity of the sample, respectively, $H=h+4\varepsilon \sigma T^{3}$, h is the convection coefficient and $4\varepsilon \sigma T^{3}$ is the heat radiation exchange term. According to $4\varepsilon \sigma T^{3}\ll h$ [30], the heat radiation exchange term can be neglected. Subscripts behind the comma mean the calculation of partial derivatives for the corresponding subscripts. The endothermic source in the sample is Q(z,t), which can be expressed as [28]:

(3) $$Q(z,t)=\beta (1-\beta^{\prime })w_{p}\exp(-\beta z)[1+\exp(j\omega t)],$$

where β is the bulk absorption coefficient of the sample, $\beta^{\prime }$ is the surface absorption rate of the sample, ω is the modulation angular frequency, and $K_{s}$, $K_{g}$ is the thermal conductivity of the sample and air.

In linear theory, $K_{s}$ is generally assumed to be constant; however, when the local temperature rise is significantly higher than the average temperature, $K_{s}$ this assumption is not valid. Therefore, this paper introduces nonlinear thermal conductivity coefficients into the nonlinear thermal diffusion equation to investigate the theory of nonlinear photothermal imaging.

In nonlinear thermal diffusion theory, the dependence of thermal conductivity on the temperature is usually considered to be linear to simplify the problem [31]:

(4) $$K_{s}=K_{0}[1+bT_{s}(z,t)],$$

where $K_{0}$ is the thermal conductivity of the sample at steady−state temperature, b is the temperature coefficient of thermal conductivity, and in general, b≪1 [32]. Substituting Equation (4) into Equation (1), the one−dimensional nonlinear thermal diffusion equation is obtained as follows:

(5) $$T_{s,zz}(z,t)=\frac{1}{\alpha_{b}}T_{s,t}(z,t)-bT_{s}(z,t)T_{s,zz}(z,t)-b{\left[T_{s,z}(z,t)\right]}^{2}-\frac{Q(z,t)}{K_{0}},$$

where $\alpha_{b}=K_{0}/(\rho C_{P}).$ Subscripts z and t behind the comma mean the calculation of partial derivatives for the corresponding subscripts.

### 2.3. Iterative Numerical Method for Solving the Nonlinear Heat Diffusion Equation

Because it is difficult to obtain a general solution of the one−dimensional nonlinear thermal diffusion equation, a new numerical method is proposed in this paper as follows. In many cases, it is advantageous to separate the variables t and z in T(z,t). In general, the Fourier series expansion T(z,t) in the frequency domain can be expressed as [33]:

(6) $$T_{s}(z,t)=A_{0}/2+\sum_{n=1}^{\infty }\left(1/2\right)A_{n}\exp\left(jn\omega t\right)+\sum_{n=1}^{\infty }\left(1/2\right)A_{n}^{*}\exp\left(-jn\omega t\right),$$

where j is an imaginary unit, $A_{0}$ is a real field variable, and $A_{0}/2$ represents the DC component of the sound wave. When $A_{n}(n\geq 1)$ is the complex field variable (i.e., complex amplitude) of the nth−order harmonic, the real part of $A_{n}\exp(jn\omega t)$ is the real displacement of the nth-order harmonic, and $A_{n}^{*}(n\geq 1)$ is the complex conjugate field variable of $A_{n}$. Note that $A_{n}$ and $A_{n}^{*}$ no longer contain the time variable t, which is a function of the spatial coordinate z.

Generally, higher−order harmonics are weaker, and some higher−order harmonics can be ignored according to the requirements for calculation accuracy. To simplify the theoretical description, only harmonics of the order less than or equal to N(N≤5) are considered in this paper, and other higher−order harmonics are ignored; this is referred to as an N−order approximation.

Substituting Equation (6) into Equation (5), the following equation can be obtained by orthogonality:

(7) $$A_{i,zz}-i\omega \alpha_{b}^{-1}A_{i}j=-\frac{b}{2}F_{i},(i=0,\pm 1,\pm 2,\pm 3,\pm 4,\pm 5),$$

where,

(8) $$\begin{array}{l} F_{0}=A_{0}A_{0,zz}+A_{1}^{*}A_{1,zz}+A_{2}^{*}A_{2,zz}+A_{3}^{*}A_{3,zz}+A_{4}^{*}A_{4,zz}+A_{5}^{*}A_{5,zz}+A_{1}A_{1,zz}^{*}+A_{2}A_{2,zz}^{*}+A_{3}A_{3,zz}^{*}\\ +A_{4}A_{4,zz}^{*}+A_{5}A_{5,zz}^{*}+A_{0,z}A_{0,z}+2A_{1,z}A_{1,z}^{*}+2A_{2,z}A_{2,z}^{*}+2A_{3,z}A_{3,z}^{*}+2A_{4,z}A_{4,z}^{*}+2A_{5,z}A_{5,z}^{*}, \end{array}$$

(9) $$\begin{array}{l} F_{1}=A_{0,zz}A_{1}+A_{1,zz}A_{0}+A_{1}^{*}A_{2,zz}+A_{2}^{*}A_{3,zz}+A_{3}^{*}A_{4,zz}+A_{4}^{*}A_{5,zz}+A_{1,zz}^{*}A_{2}+A_{2,zz}^{*}A_{3}+A_{3,zz}^{*}A_{4}+\\ A_{4,zz}^{*}A_{5}+A_{0,z}A_{1,z}+2A_{2,z}A_{1,z}^{*}+2A_{3,z}A_{2,z}^{*}+2A_{4,z}A_{3,z}^{*}+2A_{5,z}A_{4,z}^{*}\\ -\beta (1-\beta^{\prime })w_{P}\exp(-\beta z)/K_{0}, \end{array}$$

(10) $$\begin{array}{l} F_{2}=A_{0,zz}A_{2}+A_{1,zz}A_{1}+A_{2,zz}A_{0}+A_{3,zz}A_{1}^{*}+A_{4,zz}A_{2}^{*}+A_{5,zz}A_{3}^{*}+A_{1,zz}^{*}A_{3}+A_{2,zz}^{*}A_{4}+A_{3,zz}^{*}A_{5}+\\ A_{1,z}A_{1,z}+2A_{2,z}A_{0,z}+2A_{3,z}A_{1,z}^{*}+2A_{4,z}A_{2,z}^{*}+2A_{5,z}A_{3,z}^{*}, \end{array}$$

(11) $$\begin{array}{l} F_{3}=A_{0,zz}A_{3}+A_{1,zz}A_{2}+A_{2,zz}A_{1}+A_{3,zz}A_{0}+A_{4,zz}A_{1}^{*}+A_{5,zz}A_{2}^{*}+A_{1,zz}^{*}A_{4}+A_{2,zz}^{*}A_{5}+2A_{0,z}A_{3,z}+\\ 2A_{1,z}A_{2,z}+2A_{2,z}A_{1,z}+2A_{1,z}^{*}A_{4,z}+2A_{2,z}^{*}A_{5,z}, \end{array}$$

(12) $$\begin{array}{l} F_{4}=A_{0,zz}A_{4}+A_{1,zz}A_{3}+A_{2,zz}A_{2}+A_{3,zz}A_{1}+A_{4,zz}A_{0}+A_{5,zz}A_{1}^{*}+A_{1,zz}^{*}A_{5}+2A_{0,z}A_{4,z}+2A_{1,z}A_{3,z}+\\ 2A_{2,z}A_{2,z}+2A_{3,z}A_{1,z}+2A_{4,z}A_{0,z}+2A_{5,z}A_{1,z}^{*}, \end{array}$$

(13) $$F_{5}=A_{0,zz}A_{5}+A_{1,zz}A_{4}+A_{2,zz}A_{3}+A_{3,zz}A_{2}+A_{4,zz}A_{1}+A_{5,zz}A_{0}+2A_{0,z}A_{5,z}+2A_{1,z}A_{4,z}+2A_{2,z}A_{3,z}.$$

Notably, Equation (7) gives only an equation of field variables and not an equation of conjugate field variables. The equation of conjugate field variables can be obtained by taking the complex conjugate of Equation (7); therefore, Equation (7) is complete. Substituting i=0,±1,±2,±3,±4,±5 into Equation (7) yields a set of nonlinear equations. However, it is difficult to solve these equations directly. Therefore, a simple iterative method for solving these equation is proposed, where $A^{*(m)}$ and $A^{\left(m\right)}$ denotes the field quantities obtained from the m−th iterative calculation. In the iterative calculation, the following method is used. First, the field quantities on the left side of Equation (7) are taken as $A_{i}^{\left(m\right)}$ and the constants on the right side are taken as $A_{i}^{\left(m-1\right)}$ and $A_{i}^{*(m-1)}$. Second, $A_{i}$ and $A_{i}^{*}$ in $F_{i}$ are replaced with $A_{i}^{\left(m-1\right)}$ and $A_{i}^{*(m-1)}$, respectively. Third, the result obtained is $F_{i}^{\left(m-1\right)}$. Therefore, the following equation is used in the m−th iterative calculation:

(14) $$A_{i,zz}^{\left(m\right)}-i\omega \alpha_{b}^{-1}A_{i}^{\left(m\right)}j=-\frac{b}{2}F_{i}^{\left(m-1\right)},$$

when i=0,±1,±2,±3,±4,±5, an uncoupled set of equations can be obtained from (14). Therefore, $A_{i}^{\left(m\right)}$ can be calculated separately and independently from the other iterations, which means that the computational effort does not increase dramatically when higher-order harmonics are involved.

Outputting results $A_{i}^{\left(m\right)}$ of the 1 iteration calculation are same with those predicted by the linear approximation theory. Outputting results $A_{i}^{\left(2\right)}$(i≠1) of the 2 iteration calculation are same with those predicted by the perturbation theory. The depletion of pump waves has already been taken into account in the 2 interaction calculation. However, it is not taken into account in the perturbation theory [31].

In the iterative calculation, this study let $A_{i}^{\left(0\right)}=0$ and $A_{i}^{*(0)}=0$, and this paper uses the boundary excitation conditions to generate nonzero values of $A_{i}^{\left(m\right)}$ and $A_{i}^{*(m)}$. At both endpoints z=0 and z=d, this work considers that the photothermal radiation signal is mainly due to the alternating temperature field. To simplify the calculation, this paper ignores the DC term and the effect of other layers and convective radiation on the temperature

## 3. Numerical Results and Discussion

In this section, we numerically calculate the solution to Equation (5) using the new numerical iterative method and the conventional perturbation method and discuss the effect of different sample parameters on the amplitude of the posterior surface of the sample. Table 1 lists the physical parameters used in the calculation of the PWS samples. The thermal conductivity is $K_{0}=121.5$ W/m and the thermal diffusion coefficient is $\alpha_{b}=5.9419\times 10^{-5}$ m^2^/s for the 2219 aluminum alloy sample.

**Figure 2:**
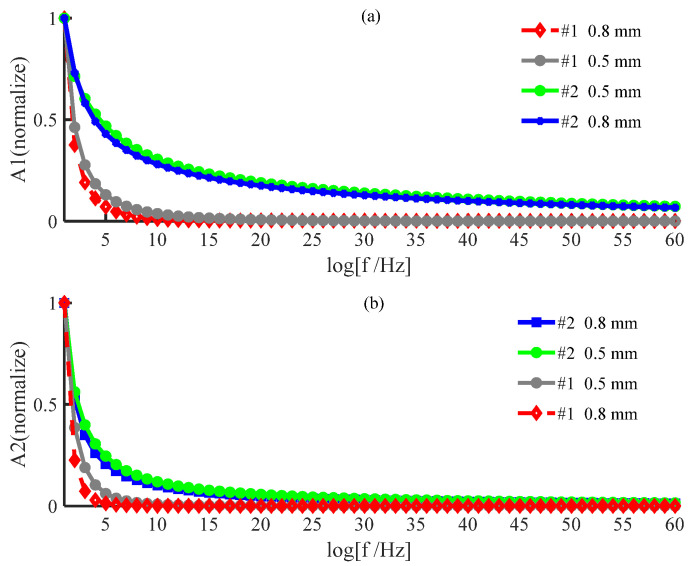
Variation in the amplitude with frequency on the posterior surface of a wine−discolored sample when the sample thickness varies: (**a**) fundamental frequency wave; (**b**) second harmonic (low frequency).

**Figure 3:**
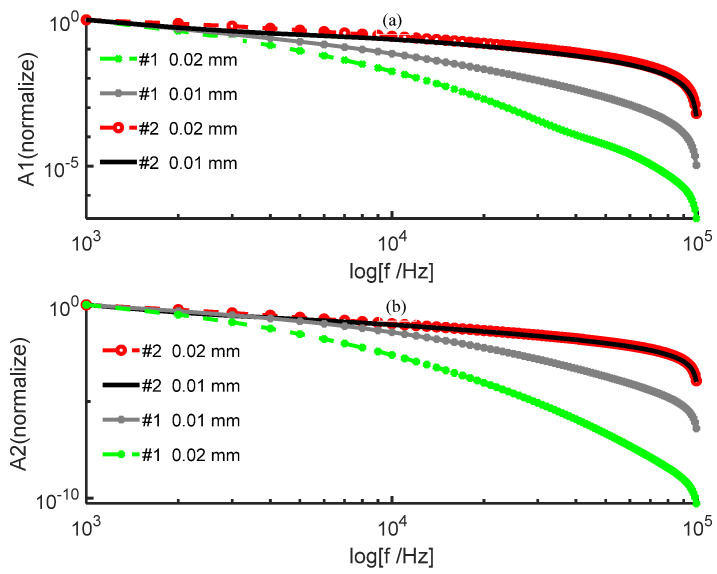
Variation in the amplitude of the posterior surface of the wine−discolored samples with frequency for different sample thicknesses: (**a**) fundamental frequency wave; (**b**) second harmonic (high frequency).

The amplitude of the signal from the posterior surface of PWS samples of different thicknesses obtained by the two numerical methods decreases with increasing frequency in the low−frequency and high−frequency ranges as shown in Figure 2 and Figure 3, respectively. In the low−frequency range, Figure 2 shows that the amplitude of each order harmonic of the signal from the posterior surface of the PWS samples obtained by both numerical methods decreases with increasing frequency when other parameters are constant and the sample thicknesses are d=0.5 mm and d=0.8 mm. In the high−frequency range, Figure 3 reveals that the amplitude of each order harmonic of the signal from the posterior surface of the PWS samples obtained by both numerical methods decreases with increasing frequency when other parameters are constant and the thickness of the samples is d=0.01 mm and d=0.02 mm.

As seen in Figure 2 and Figure 3, the results obtained by the new numerical iterative method are more sensitive to thickness than those obtained by the conventional perturbation method. Figure 2 shows the results in the low−frequency range PWS samples with thicknesses d=0.5 mm and d=0.8 mm. Both the fundamental and second harmonics on the posterior surface of the samples obtained by the two numerical methods decrease with increasing thickness, which is in good agreement with the results predicted in the literature [28,29]. For the fundamental and second harmonics obtained by the conventional perturbation method, there is no significant change in the effect of the sample thickness on the results, and there is no significant difference between the fundamental and second harmonics. However, the fundamental and second harmonics obtained by the new numerical method have a statistically significant effect on the results due to the change in the sample thickness, and the change is more pronounced in the second harmonic, so that it is necessary to consider the second harmonic. Figure 3 shows the results in the high-frequency range for the PWS samples with the thicknesses of d=0.01 mm and d=0.02 mm. Both fundamental frequency waves and second harmonics on the posterior surface of the samples obtained by the new method decrease with increasing thickness. However, both fundamental frequency waves and second harmonics on the posterior surface of the samples obtained by the conventional perturbation method increase with thickness, contradicting the literature predictions. Therefore, it is possible that the conventional perturbation method is not applicable at high frequencies. Additionally, the new numerical method shows a more pronounced change in the second harmonic than in the fundamental frequency wave when a weak change in the sample thickness occurs. Therefore, the second harmonic has stronger sensitivity to thickness, and the new method has the potential for important applications in the noninvasive assessment of PWS thickness.

Reference [31] applied the perturbation method to the solution of the nonlinear thermal diffusion equation to theoretically study the nonlinear photoacoustic effect; while the physical meaning of its simple method and solution is clear, Reference [31] considers only the effect of lower−order harmonics on higher−order harmonics and ignores the inverse effect. Figure 2 and Figure 3 show that the sensitivity of the perturbation method to the sample thickness and the applicable frequency range is slightly lower effective than that of the new method.

**Figure 4:**
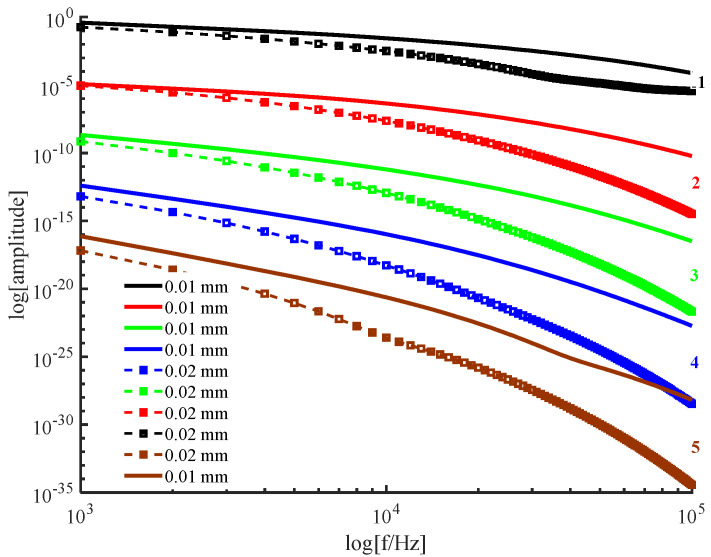
Variation in the harmonic amplitude with frequency for each order of harmonic for different sample thicknesses.

In Figure 4, the solid line shows the thickness of the PWS sample inv d=0.01 mm, and the marked dashed line shows the thickness of the PWS sample in d=0.02 mm. The numbers 1−5 indicate the fundamental frequency wave and second through fifth harmonics, respectively.

Figure 4 shows the variation in the amplitude of each order of harmonics the frequency on the posterior surface of PWS samples at different thicknesses obtained by the new method. From Figure 4, the following conclusion can be drawnwhile other parameters held constant. First, each order of harmonic decreases with increasing frequency. Second, each order of harmonic decreases with increasing thickness. Third, the rate of change with thickness is larger for higher−order harmonics than lower−order harmonics, indicating that the effects of higher−order harmonics are more sensitive to the change in the sample thickness than those of the lower−order harmonics. Therefore, it is necessary to consider higher−order harmonics. In addition, the difference between the rates of change of the fourth and fifth harmonics with thickness is not very obvious. Therefore, the choice N≤5 is appropriate in the theoretical derivation.

**Figure 5:**
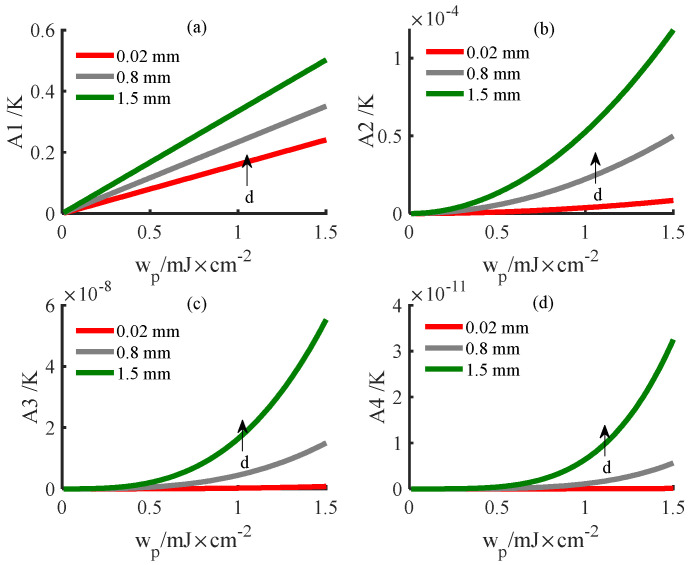
Variation in the harmonic amplitude with light energy for different sample thicknesses. (**a**) Fundamental frequency wave; (**b**) second harmonic; (**c**) third harmonic; (**d**) fourth harmonic.

Figure 5 shows the variation of each order of harmonics with light energy on the posterior surface of the PWS samples at different thicknesses obtained with the new method. Figure 5 shows that the amplitude of each order of harmonics increases with increasing light energy when other parameters are constant. Additionally, when other parameters are constant, the amplitude of each order of harmonic decreases with increasing thickness. As observed from Figure 5a, the fundamental frequency amplitude is proportional to the optical energy $w_{p}$, which is consistent with the results of linear theory. As observed from Figure 5b,c, the amplitudes of the higher−order harmonics are proportional to the square of the light energy, which is consistent with the theoretical derivation.

To demonstrate the validity of the new theory, two numerical methods were used to analyze and compare the results for the variation in the amplitude with sample thickness on the posterior surface of 2219 aluminum alloy samples. The two methods are the new iterative numerical method and the conventional perturbation method [28,29].

**Figure 6:**
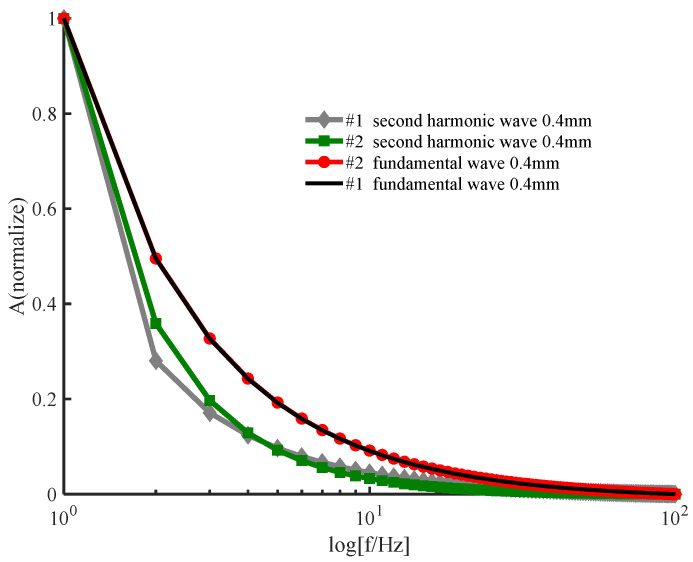
Variation in the fundamental frequency wave and second harmonic with frequency.

**Figure 7:**
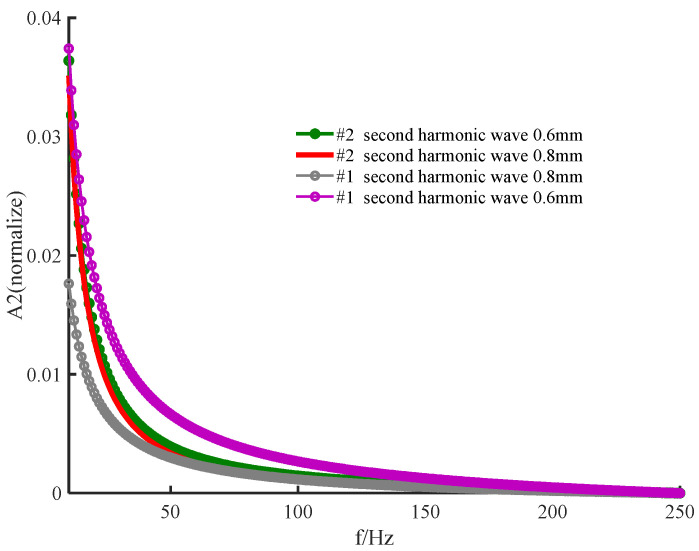
Variation in the second harmonic with frequency for different sample thicknesses.

The effectiveness of the new iterative numerical method for solving the nonlinear heat diffusion equation is demonstrated in Figure 6 and Figure 7. Numerical simulations of the nonlinear heat diffusion equation for the 2219 aluminum alloy sample using the new numerical iterative method show two effects. First, the amplitude of each order of harmonics decreases with increasing frequency. Second, the second−order harmonic amplitude decreases with increasing thickness. The above conclusions are consistent with those obtained by the conventional perturbation method, and Figure 6 shows that the fundamental frequency wave results obtained by the two methods are very consistent. Therefore, the effectiveness of the method is demonstrated in Figure 6 and Figure 7.

An examination of Figure 6 and Figure 7 shows the superiority of the new numerical method. For the 2219 aluminum alloy sample, the rate of change with the frequency of the second harmonic is larger than that of the fundamental frequency wave, and the rate of change of the results obtained by the new numerical method is larger than that of the second harmonic results obtained by the traditional perturbation method. Additionally, Figure 7 shows that the rate of change of the second harmonic amplitude with increasing thickness with other parameters unchanged is greater with the new method than with the traditional method. Therefore, the new numerical method is better than the traditional perturbation method. This comparison reflects the greater sensitivity of the results to the sample thickness obtained by the proposed method compared tothe traditional method.

**Figure 8:**
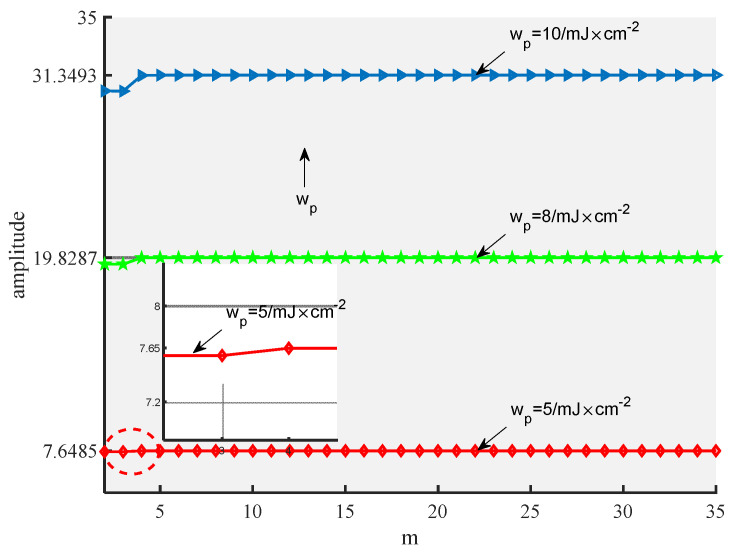
The variation in the second harmonic amplitude with number of iterations for different light energies.

Figure 8 describes the relationship between the second harmonic amplitude on the posterior surface of the 2219 aluminum alloy sample and the iteration frequency m when the light energy $w_{p}$ differs. Figure 8 shows that when the light energy is 5 mJ/cm^2^ and the number of iterations is greater than or equal to 6, the fundamental frequency amplitude converges to 7.6485 K. In addition, when the light energy is 8 mJ/cm^2^ and the number of iterations is greater than or equal to 10, the fundamental frequency amplitude converges to 19.8287 K. Moreover, when the light energy is 10 mJ/cm^2^ and the number of iterations is greater than or equal to 12, the fundamental frequency amplitude converges to 31.3493 K. These results imply that the proposed method is converges well.

## 4. Conclusions

In this paper, a theoretical model for thermal nonlinear photoacoustic detection related to port-wine stain samples is constructed. A new numerical iteration approach is developed and compared to the perturbation method for analyzing the thickness and absorption coefficient of PWS samples. The main conclusions are as follows:

(1) The rates of change with frequency, thickness, and optical energy intensity are larger for higher−order harmonics than lower-order harmonics; higher−order harmonics are more sensitive to sample detection than lower-order harmonics.
(2) For the same parameter values, the proposed new numerical iterative method has greater sensitivity and a wider frequency band than the perturbation method. Furthermore, the calculation time of our proposed method will not drastically increase when additional high−order harmonics are included.

## Figures and Tables

**Table 1:** Thickness, optical and thermal parameters of the skin model at 840 nm [11,25,26,27].

Layers	d (mm)	β (mm^−1^)	σ (mm^−1^)	g	ρ (g/cm^−3^)	C (J/(g. K))	K_0_ (mW/(cm. K))
Stratum corneum	0.01	0.00091	18.95	0.8	1.2	3.59	2.4
Living epidermis	0.08	0.13	18.95	0.8	1.2	3.59	2.4
Papillary dermis	0.10	0.105	11.65	0.8	1.09	3.35	4.2
Upper blood plexus	0.08	0.15875	15.485	0.818	1.09	3.35	4.2
Reticular dermis	1.50	0.105	11.65	0.8	1.09	3.35	4.2
Deep blood plexus	0.07	0.4443	46.165	0.962	1.09	3.35	4.2
Dermis	0.16	0.105	11.65	0.8	1.09	3.35	4.2
Hypodermis	3.00	0.009	11.44	0.9	1.21	2.24	1.97
Muscle tissues	3.00	0.029	7.13	0.9	1.075	3.5	4.5
PWS	0.001~1.5	0.15875	46.7	0.99	1.0	3.6	5.3

## Data Availability

The data that support the findings of this study are available from the corresponding author (H.C.), upon reasonable request.
